# Scarce Duet in One Heartbeat: A Case Report on Yamaguchi and Myocardial Bridging

**DOI:** 10.7759/cureus.104531

**Published:** 2026-03-02

**Authors:** Nada Saleh, Lama El Mawla, Zeinab Shaito, Ahmad H Alhajj Mohammad, Majd Khalil

**Affiliations:** 1 Internal Medicine, Lebanese University Faculty of Medicine, Beirut, LBN; 2 Cardiology, Lebanese University Faculty of Medicine, Beirut, LBN

**Keywords:** ace of spades, apical hypertrophy, cardiology, hypertrophic cardiomyopathy, myocardial bridge, yamaguchi

## Abstract

Among the various types of hypertrophic cardiomyopathies (HCM) is an apical hypertrophic cardiomyopathy (ApHCM) variant, also known as Yamaguchi syndrome. It is a rare condition mostly found in Japanese patients and is characterized by the special Ace-of-Spades morphology of the left ventricular cavity. The coexistence of ApHCM and myocardial bridging is an uncommon finding. This rare association may create a complex arrhythmogenic and ischemic substrate, potentially increasing the risk of adverse cardiac events.

In our patient, echocardiography demonstrated an apical wall thickness of 17 mm, a left ventricular outflow tract (LVOT) maximum peak gradient of 3.45 mmHg, and a LVOT mean gradient of 2.47 mmHg, along with a global longitudinal strain (GLS) value of −15.8%, consistent with a non-obstructive LVOT physiology.

While its symptoms coincide with those of acute coronary syndrome (ACS), clinicians should maintain a high index of suspicion for this disease, as it is often unrecognized. This paper documents the rare coexistence of Yamaguchi syndrome and myocardial bridging in a patient with a previous history of cardiac arrest, highlighting its rarity, discussing the diagnostic challenges due to symptoms mimicking acute coronary syndrome (ACS), and emphasizing the importance of careful management and follow-up in affected patients.

## Introduction

Yamaguchi syndrome, or apical hypertrophic cardiomyopathy (ApHCM), is a type of cardiomyopathy that involves the apical region of the left ventricle. ApHCM is less explored in terms of its associated diagnosis and long-term outcomes than HCM, thus remaining a diagnostic challenge. This variant is often misidentified because its symptoms resemble those of acute coronary syndrome [1]. It is a rare entity of hypertrophic cardiomyopathy (HCM), characterized by the absence of left ventricular outflow tract (LVOT) obstruction, and patients may be asymptomatic or present with shortness of breath, chest discomfort, palpitations, fatigue, syncope, or sudden cardiac death [2]. The formal diagnostic criteria for apical hypertrophic cardiomyopathy, in accordance with European Society of Cardiology (ESC) [3] and American Heart Association (AHA) [4] guidelines, include an apical wall thickness ≥15 mm and an apical-to-posterior wall thickness ratio ≥1.5.

Myocardial bridging (MB) refers to a condition in which a coronary artery extends beyond its normal epicardial domain and becomes embedded in the myocardium. It is considered an unusual yet harmless variant [5]. Myocardial bridging was first described by Henric Reyman in 1732 during an autopsy as a tunneled segment of the coronary artery [5]. MB is a well-recognized phenomenon with a prevalence of 1-3% in the general population, but it is significantly more frequent in patients with HCM, with prevalence rates increasing to 30% [6].

We present a case of Yamaguchi syndrome associated with myocardial bridging, tackling the diagnostic modalities and emphasizing its clinical importance, as it is frequently underdiagnosed or discovery is delayed.

## Case presentation

We present a case of a 50-year-old Lebanese man, a non-smoker, with no known food or drug allergies and a medical history significant for hypertension, dyslipidemia, and recurrent nephrolithiasis. His past surgical history includes the placement of a double-J ureteral stent for a previous obstructing kidney stone. He presented to our institution for double-J ureteral stent insertion for nephrolithiasis.

During the pre-operative cardiac consultation, the patient reported a history of cardiac arrest that occurred during a previous procedure (double-J ureteral stent insertion) two years ago. Details regarding the event, including rhythm documentation, duration of cardiopulmonary resuscitation (CPR), and defibrillation requirements, were unavailable, as it occurred two years ago at another institution. Troponin levels from that episode were also not accessible. Investigation at that time included a trans-thoracic echocardiography (TTE) that was reported as normal with an ejection fraction (EF) of 70%, and a cardiac catheterization that ruled out occlusive coronary arterial disease but showed severe myocardial bridging. Coronary angiography demonstrated systolic compression of the central section of the left anterior descending (LAD) artery, consistent with myocardial bridging. Angiographic images were not available for inclusion, and a quantitative assessment of the myocardial bridging, including the percentage of systolic narrowing, could not be performed, which represents a limitation of this report.

**Figure 1:**
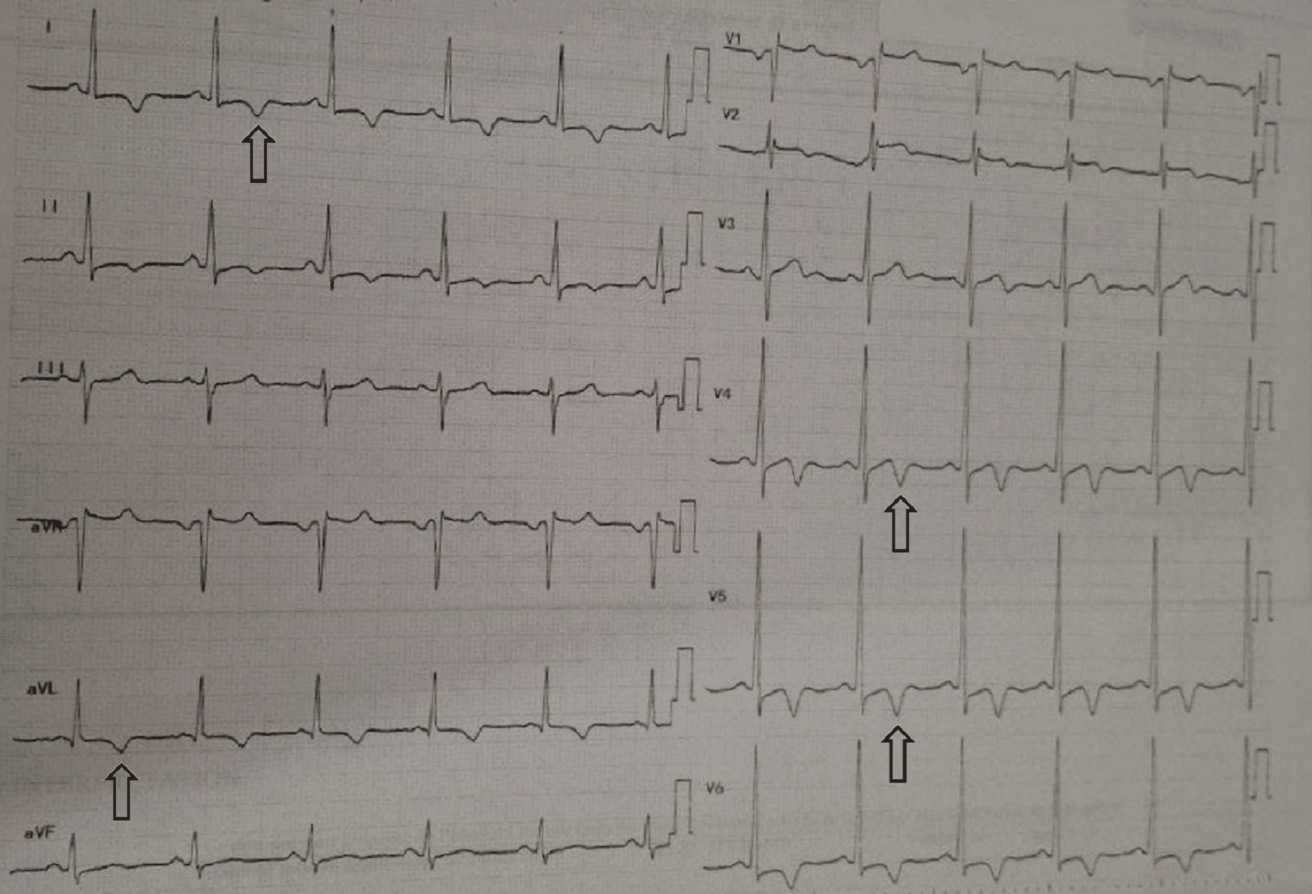
ECG showing T wave inversion in multiple precordial and limb leads ECG: electrocardiogram

An electrocardiogram (ECG) at our hospital showed a pattern of left ventricular hypertrophy (LVH) and inverted T waves in precordial and limb leads, indicating left ventricular strain (Figure 1). ECG showed deep T‑wave inversions of 9 mm, evidence of left ventricular hypertrophy by Sokolow‑Lyon criteria (S wave in V1 + R wave in V5 = 10 + 32 = 42 mm, exceeding the 35 mm threshold), a QT interval of 400 ms, and no arrhythmias were observed.

While myocardial bridging was initially suspected as the cause of cardiac arrest, additional diagnostic evaluation was pursued based on the patient’s history and ECG findings to assess alternative etiologies.

**Figure 2:**
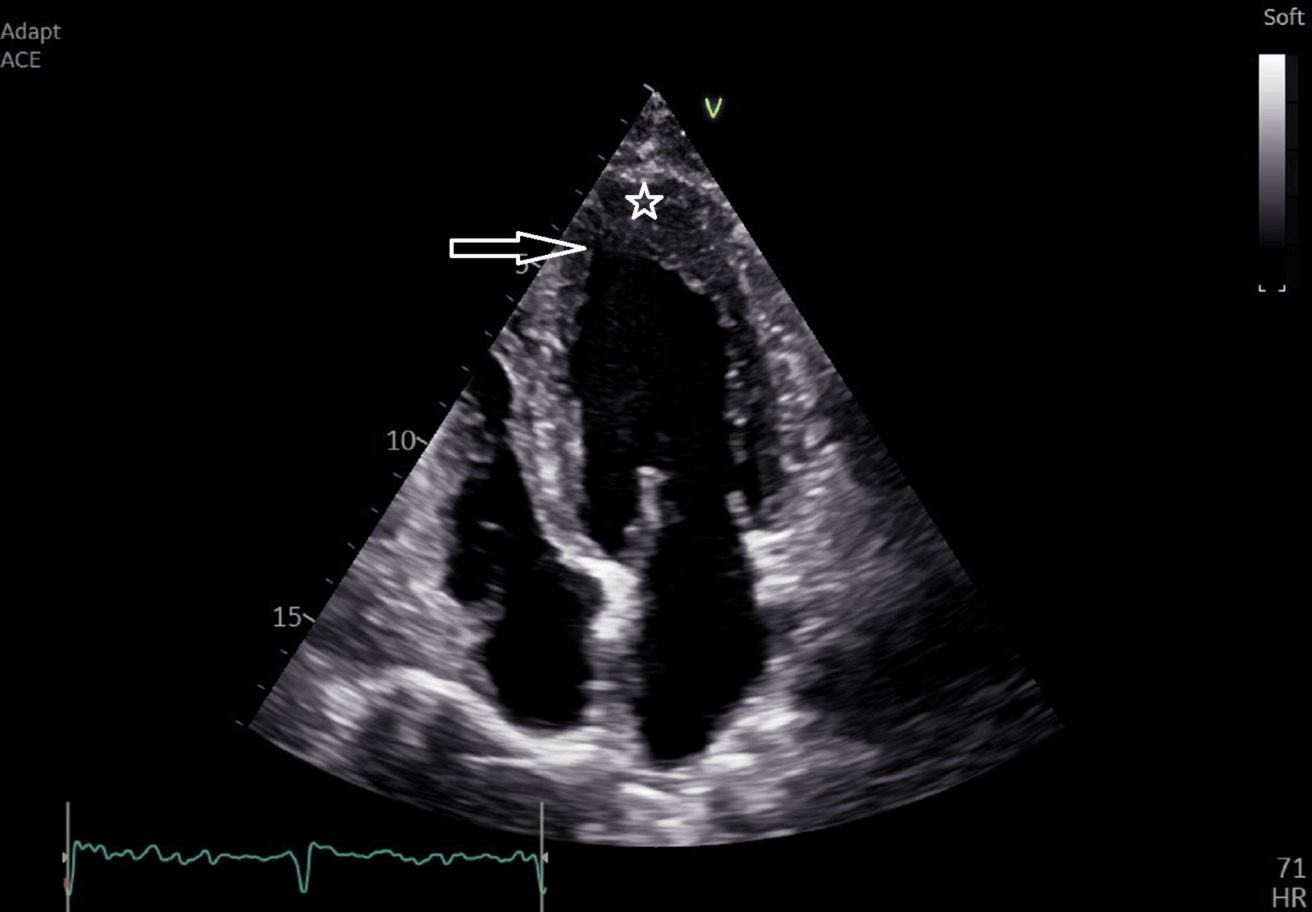
TTE showing apical hypertrophy (star) with the Ace-of-Spades configuration (arrow) TTE: trans-thoracic echocardiography

TTE was ordered as a pre-request for cardiac clearance prior to surgery (Figure 2). It revealed a left ventricle with a normal chamber size and preserved systolic function, with an estimated ejection fraction of 67%.

**Figure 3:**
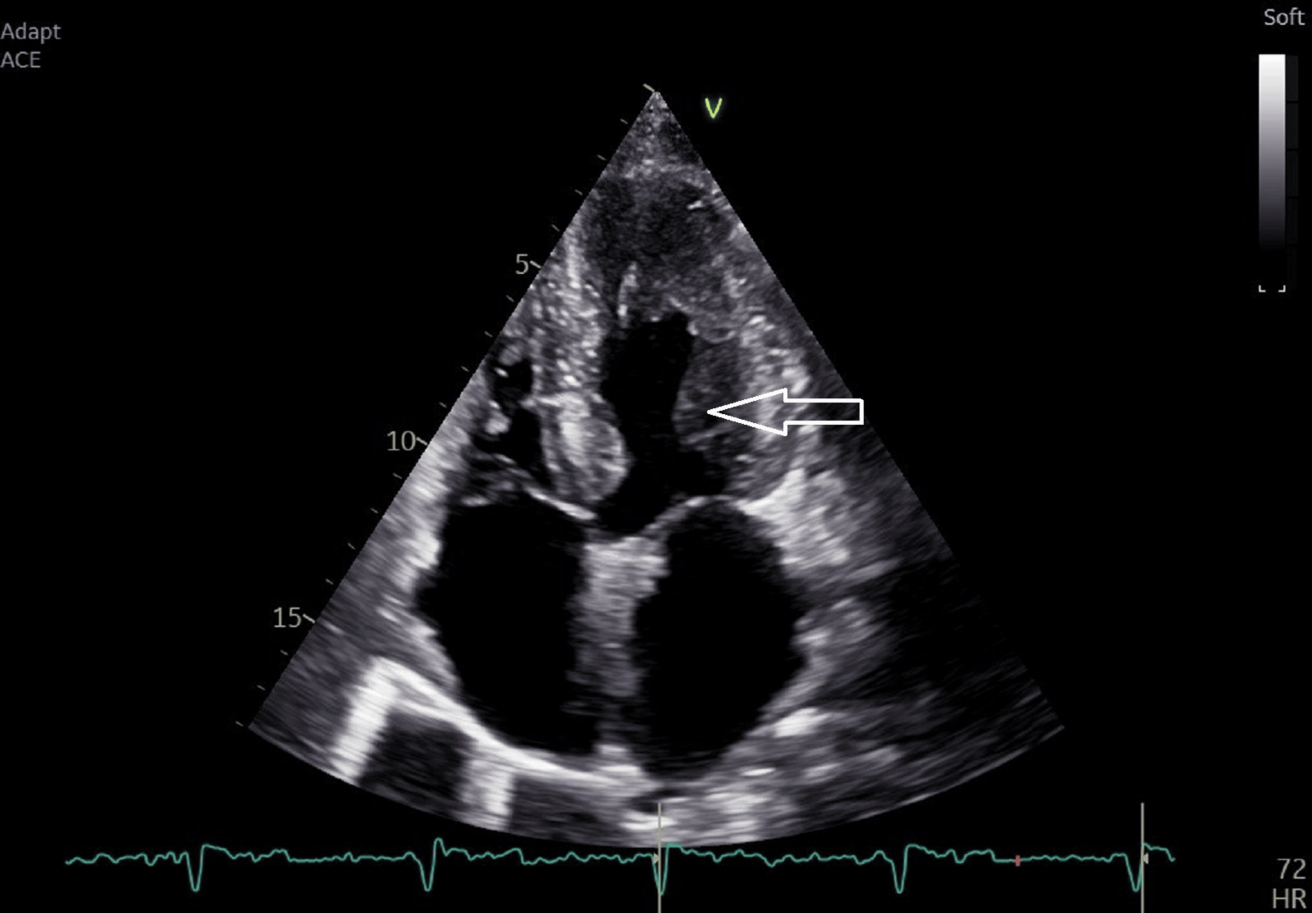
TTE showing the Ace-of-Spades configuration with papillary muscle hypertrophy TTE: trans-thoracic echocardiography

The left ventricular wall demonstrated extensive apical hypertrophy with a characteristic Ace-of-Spades configuration (Figure 3). Echocardiography also revealed an apical wall thickness of 17 mm, a left ventricular mass index (2D method) of 110 g/m², an E/A ratio of 1.23, an E/e′ ratio of 8.64, and a normal left atrial area of 18 cm².

**Figure 4:**
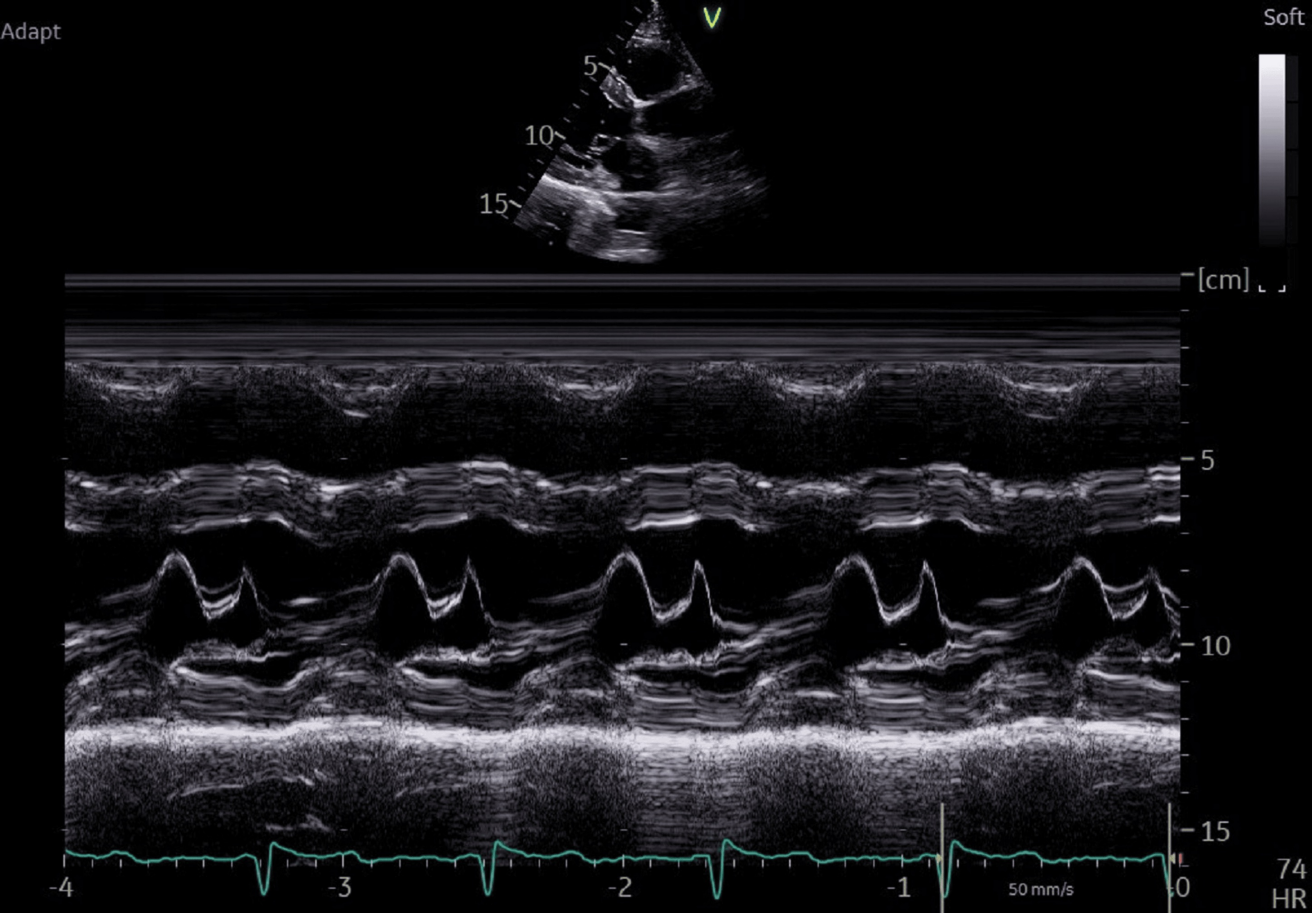
TTE showing an absence of SAM of the mitral valve, which means an absence of LVOT obstruction TTE: trans-thoracic echocardiography, SAM: systolic anterior motion, LVOT: left ventricle outflow tract

**Figure 5:**
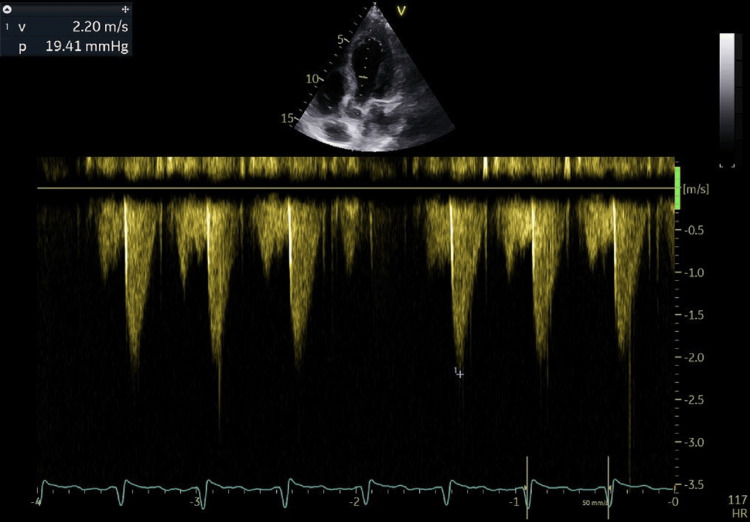
TTE showing the absence of an LV intracavitary gradient following exercise TTE: trans-thoracic echocardiography, LV: left ventricular

There was no evidence of sub-valvular obstruction or systolic anterior motion (SAM) of the mitral valve (Figure 4), and the LV inta-cavitary gradient reached a maximum of 19.4 mmHg following provocation with exertion (Figure 5).

**Figure 6:**
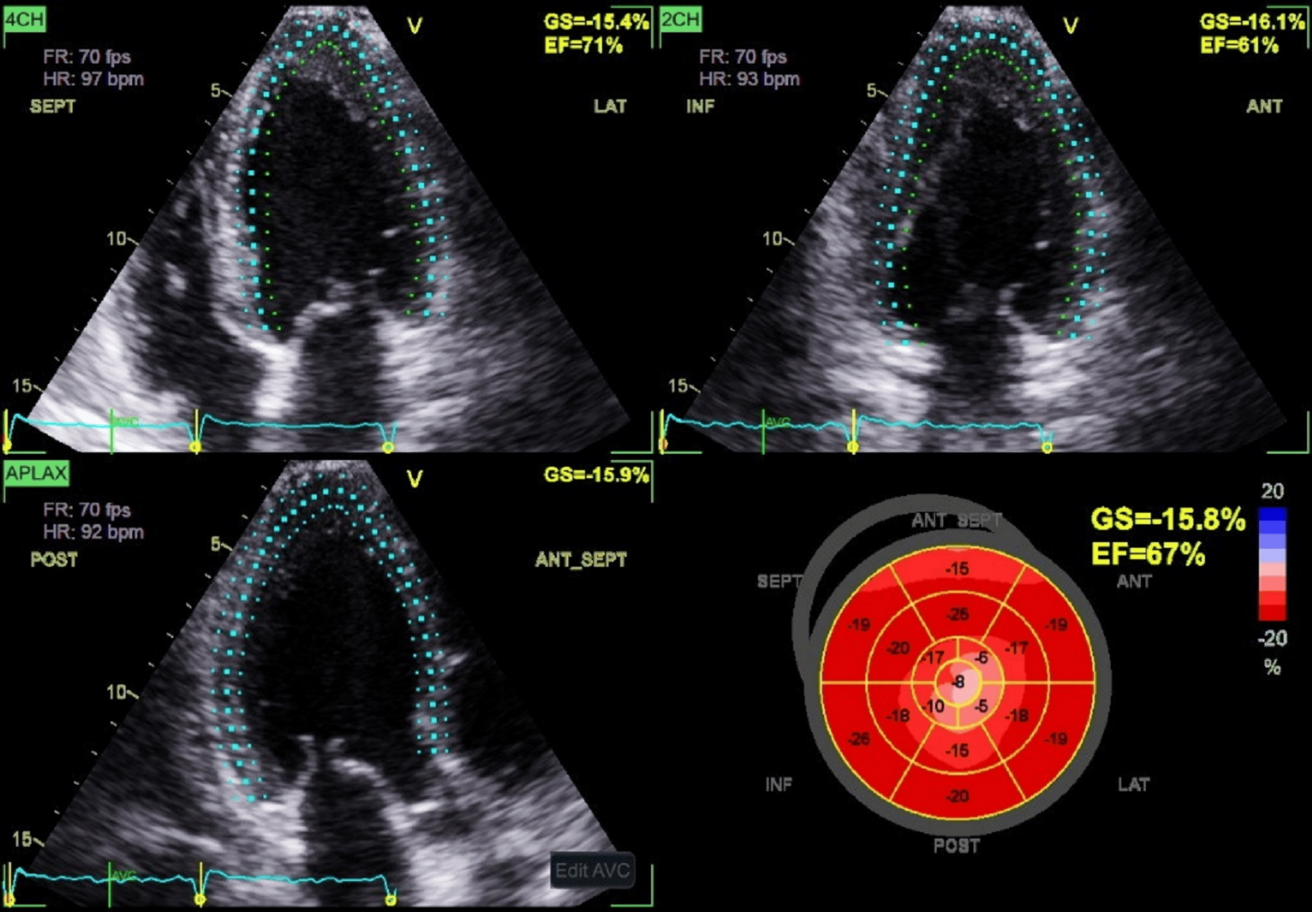
TTE showing marked reduction of the GLS at the apex TEE: trans-thoracic echocardiography, GLS: global longitudinal strain

Global longitudinal strain (GLS) revealed a marked decline of deformations confined to the apex with an average GLS of -15.8% (Figure 6). In the general population, a GLS cutoff of −16.5% predicted significant myocardial fibrosis with 80.9% sensitivity and 76.5% specificity. In patients with hypertrophic cardiomyopathy, the same cutoff demonstrated 80.9% sensitivity and 57.1% specificity. GLS also served as an independent predictor of myocardial fibrosis and correlated with both fibrosis severity and the five-year sudden cardiac death risk score, offering important prognostic information.

Cardiac magnetic resonance imaging (MRI) was requested; however, the patient declined the examination due to claustrophobia. Preoperative laboratory studies are shown in Table 1.

**Table 1:** Laboratory tests

Test	Result	Reference range
Complete blood count		
White blood cell count	4.480 x 10˄9/L	4.0-11.0 x 10˄9/L
Hemoglobin	14 g/dL	13.5-17.5 g/dL
Hematocrit	41.80%	41-53%
Platelet count	229 x 10˄9/L	150-400 x 10˄9/L
Neutrophils	62%	40-70%
Lymphocytes	28%	20-45%
Biochemistry panel		
Creatinine	1.37 mg/dL	0.6-1.3 mg/dl
Sodium	140 mmol/L	135-145 mmol/L
Potassium	4.14 mmol/L	3.5-5 mmol/L
Chloride	101.5 mmol/L	96-106 mmol/L
Bicarbonate	26.3 mmol/L	22-29 mmol/L

The patient successfully underwent insertion of a double-J ureteral stent without any peri-procedural complications. He was discharged on a low-dose beta-blocker regimen (atenolol 25 mg once daily).

Genetic testing was recommended to evaluate for mutations commonly associated with apical hypertrophic cardiomyopathy, including ACTC1, TPM1, MYBPC3, and MYH7. Ambulatory 48-hour Holter monitoring was advised to screen for potential arrhythmias.

Cardiac magnetic resonance imaging (MRI) was strongly recommended to assess for myocardial fibrosis and potential apical aneurysm formation; however, given the patient’s history of claustrophobia, the examination will be performed with appropriate anxiolytic premedication and monitored sedation to ensure tolerance and image quality. Annual transthoracic echocardiography (TTE) was recommended for longitudinal surveillance and assessment of disease progression.

First-degree family members were counseled to undergo screening with ECG, TTE, and genetic evaluation in accordance with current recommendations for familial hypertrophic cardiomyopathy.

## Discussion

Yamaguchi syndrome, commonly known as ApHCM, is an uncommon variant of hypertrophic cardiomyopathy characterized by predominant hypertrophy of the left ventricular apex [1]. ApHCM accounts for 25% of HCM in the Asian population and 1-10% in the non-Asian population [7]. Yamaguchi was the first to describe this syndrome and its ventriculographic features in 1979, while Sakamoto was the first to report the condition's electrocardiographic pattern and echocardiographic results in Japanese patients in 1976 [8].

Yamaguchi syndrome is an entity frequently detected in East Asian populations; nonetheless, there have been only a limited number of cases documented among African Americans [9]. The average age for the onset of ApHCM is 41.4 ± 14.5 years, with a higher prevalence in males. Approximately 54% of individuals with ApHCM experience symptoms, with chest pain being the most frequently encountered symptom, followed by palpitations, shortness of breath, and fainting. ApHCM can also be associated with serious complications, including atrial fibrillation, heart attacks, embolic incidents, ventricular fibrillation, congestive heart failure, apical aneurysm, and cardiac arrest [10]. Our patient is a 50-year-old Lebanese male (West Asian region), which corresponds to the typical age of onset for ApHCM, a condition more commonly observed in males. Common features of Yamaguchi syndrome include noticeable fourth heart sound (S4), pronounced negativity of the T wave on the ECG, particularly in the left precordial leads, and an end-diastolic “spade-like” shape of the left ventricular cavity observed on left ventriculography [11]. TTE is typically the first-line diagnostic examination; however, cardiac MRI is considered the gold standard, as it provides high-resolution images that allow a detailed assessment of left ventricular hypertrophy, particularly in regions where thickening is localized to more distal segments [12].

Our patient’s ECG showed negative T waves on left precordial leads, and TTE showed the characteristic “spade-like” shape of the left ventricle. ApHCM may clinically mimic acute coronary syndrome due to the presence of chest pain and marked electrocardiographic abnormalities, particularly deep, symmetric T-wave inversions in the precordial leads. Coronary angiography is essential to exclude significant obstructive coronary artery disease in these patients. Furthermore, left ventriculography may demonstrate the characteristic “spade-like” configuration of the left ventricular cavity during systole, a hallmark feature reflecting apical myocardial hypertrophy.

In terms of the pathophysiology of ApHCM, it may manifest with or without apical aneurysm formation, as well as with or without characteristics of midventricular obstruction with cavity obliteration (MVOCO). Our patient didn’t have an intracavitary gradient.

 In Japan, ApHCM appears to demonstrate distinct clinical characteristics compared with classical HCM, both within Japan and in other populations, where genetic inheritance patterns are better established, and symptoms are often more pronounced. The underlying mechanisms responsible for the differences in phenotypic expression of apical hypertrophy between Asian and non-Asian populations remain unclear. The majority of mutations in ApHCM occur in the sarcomere, and the inheritance pattern is autosomal dominant. Most of the gene mutations in the thick myofilaments of the heart tissue are found in myosin-binding protein C (MYBPC3) and β-myosin heavy chain (MYH7) [13].

Insertion of an implantable cardiac defibrillator in HCM is based on risk factors, including previous cardiac arrest, spontaneous sustained ventricular tachycardia (VT), spontaneous nonsustained VT, family history of SCD, unexplained syncope, left ventricular thickness greater than or equal to 30 mm, and an abnormal blood pressure response to exercise. Although earlier literature suggested uncertainty regarding ICD implantation in ApHCM, current major society guidelines, including the American College of Cardiology (ACC) [4], AHA [4], and the ESC [3], apply risk stratification and ICD recommendations to all hypertrophic cardiomyopathy phenotypes, including apical hypertrophic cardiomyopathy. Therefore, ICD placement decision in ApHCM should follow the established HCM risk assessment criteria. To note that, case reports of two patients with ApHCM who got a cardiac defibrillator implanted didn’t record any abnormal cardiac electrical activity six months after implantation, emphasizing the low risk of ApHCM becoming complicated [14].

In consequence of the demand-perfusion mismatch, LV hypertrophy can potentially result in myocardial ischemia. The ApHCM also exhibits certain characteristics of intramural coronary artery constriction with the ensuing small-vessel disease without the underlying atherosclerotic disease [15]. About 22.2 % of people with HCM have myocardial bridging, which often happens in the central and distal sections of the left anterior descending artery. ApHCM is rarely linked to myocardial bridging despite the fact that HCM and myocardial bridging are frequently associated in the literature [16].

Symptoms often linked to myocardial bridging include angina pectoris caused by the compression of the bridged segment during the contraction of the heart. Moreover, different diagnostic techniques, such as ECG, stress testing, echocardiography, and CT angiography, are crucial in confirming the existence of myocardial bridging and evaluating its clinical importance [6].

In terms of definitive treatment, the management of myocardial bridging is influenced by various factors, including the severity of symptoms, the presence of cardiovascular risk factors, and the level of myocardial ischemia. For patients with mild or asymptomatic myocardial bridges, conservative strategies, such as lifestyle changes, pharmacological treatment (beta blockers), and management of risk factors, may be utilized. However, patients with ongoing symptoms or significant myocardial ischemia may require more intensive therapeutic measures. Among the available interventional options, percutaneous coronary intervention (PCI) with stent implantation and surgical myotomy are effective methods for alleviating coronary artery compression and enhancing blood flow [5].

Although coronary angiography images were unavailable, the diagnosis of myocardial bridging in our case was confirmed by the official angiographic report and supported by clinical and echocardiographic findings. Our patient was prescribed a beta blocker given the fact that he is asymptomatic.

Although myocardial bridging was initially considered as the precipitating factor for the cardiac arrest in our patient, subsequent identification of ApHCM introduces an additional plausible arrhythmogenic substrate. Thus, a definitive causal relationship cannot be established, and the arrest may have resulted from myocardial ischemia, ventricular arrhythmia related to apical hypertrophic cardiomyopathy, or the interplay of both conditions.

## Conclusions

This case report emphasizes the importance of considering Yamaguchi syndrome in patients presenting with a left ventricular strain on ECG, while acknowledging that the condition can also be found in asymptomatic individuals. It highlights the rare coexistence of Yamaguchi syndrome with myocardial bridging. Myocardial bridging was initially suspected as the cause of cardiac arrest; however, further evaluation led to the diagnosis of apical hypertrophic cardiomyopathy as a potential alternative etiology.

Our case discusses key aspects of diagnosis, formal diagnostic criteria, management strategies, and follow-up. It also underscores the need for family screening to identify at-risk relatives. Although ApHCM generally carries a favorable long-term prognosis, early and accurate recognition is essential to optimize patient outcomes, particularly given the potential risk of arrhythmias and sudden cardiac death. Our patient was managed conservatively with pharmacologic therapy and advised to undergo follow-up with genetic testing and serial cardiac imaging.
